# *In Vivo* Tracking and Comparison of the Therapeutic Effects of MSCs and HSCs for Liver Injury

**DOI:** 10.1371/journal.pone.0062363

**Published:** 2013-04-30

**Authors:** Qiang Li, Xinmin Zhou, Yongquan Shi, Jinge Li, Linhua Zheng, Lina Cui, Jun Zhang, Lu Wang, Zheyi Han, Ying Han, Daiming Fan

**Affiliations:** State Key Laboratory of Cancer Biology, Xijing Hospital of Digestive Diseases, The Fourth Military Medical University, Xi’an, Shaanxi Province, China; Indian Institute of Toxicology Reserach, India

## Abstract

**Background:**

Mesenchymal stem cells (MSCs) and hematopoietic stem cells (HSCs) have been studied for damaged liver repair; however, the conclusions drawn regarding their homing capacity to the injured liver are conflicting. Besides, the relative utility and synergistic effects of these two cell types on the injured liver remain unclear.

**Methodology/Principal Findings:**

MSCs, HSCs and the combination of both cells were obtained from the bone marrow of male mice expressing enhanced green ﬂuorescent protein(EGFP)and injected into the female mice with or without liver fibrosis. The distribution of the stem cells, survival rates, liver function, hepatocyte regeneration, growth factors and cytokines of the recipient mice were analyzed. We found that the liver content of the EGFP-donor cells was significantly higher in the MSCs group than in the HSCs or MSCs+HSCs group. The survival rate for the MSCs group was significantly higher than that of the HSCs or MSCs+HSCs group; all surpassed the control group. After MSC-transplantation, the injured livers were maximally restored, with less collagen than the controls. The fibrotic areas had decreased to a lesser extent in the mice transplanted with HSCs or MSCs+HSCs. Compared with mice in the HSCs group, the mice that received MSCs had better improved liver function. MSCs exhibited more remarkable paracrine effects and immunomodulatory properties on hepatic stellate cells and native hepatocytes in the treatment of the liver pathology. Synergistic actions of MSCs and HSCs were most likely not observed because the stem cells in liver were detected mostly as single cells, and single MSCs are insufficient to provide a beneficial niche for HSCs.

**Conclusions/Significance:**

MSCs exhibited a greater homing capability for the injured liver and modulated fibrosis and inflammation more effectively than did HSCs. Synergistic effects of MSCs and HSCs were not observed in liver injury.

## Introduction

Liver transplantation remains the definitive treatment option for end-stage liver disease. But the mismatch between the number of patients requiring transplantation and the amount of available organs is set to grow, highlighting the need to develop new strategies to reduce liver scarring and stimulate liver regeneration. Stem cell replacement strategies are therefore being investigated as an attractive alternative approach to liver repair.

To date, there are several published human clinical studies investigating the effects of stem cell therapy in patients with liver disease and most of the studies yielded positive results. The cells mostly used to transplant were derived from bone marrow including MSCs [1], HSCs [2], [3] and unsorted mononuclear cells [4].Among them, MSCs and HSCs can be obtained in a great quantity. MSCs represent excellent candidates for the following reasons: MSCs (i) can contribute to the direct production of new hepatocytes [4], [5], [6], (ii) can promote tissue repair by secreting trophic molecules [6], [7], [8], (iii) are immunomodulatory and hypoimmunogenic (i.e., in addition to not being recognized by the recipient’s immune system unlike allogeneic transplants, MSCs could manage exacerbated inflammatory process) [9], [10], [11] and (iv) have anti-fibrotic properties and inhibit the activation of hepatic stellate cells [11], [12]. HSCs represent another significant stem cell type in bone marrow. The liver-specific phenotypic and functional changes of a highly enriched population of HSCs in response to injury in vitro or *in vivo* have been examined. HSCs also have many of the following advantages: HSCs (i) can also be converted into functional hepatocytes without fusion and therefore contribute to the regeneration of injured liver [13], (ii) can reduce liver injury through paracrine effects [4] and (iii) can modulate the immune system of the recipient [14]. However, which cell type is more effective in treating the injured liver remains to be determined. Moreover, several recent studies have suggested that MSCs and HSCs function synergistically for the therapy of diabetes and heart failure and for vascularizing bioengineered tissues [15], [16], [17]. Whether these cells can work synergistically in the injured liver is unclear.

Our aim was to evaluate the biodistribution of the stem cells after the peripheral infusion of MSCs or HSCs into liver injured mice. The anti-inflammatory and anti-fibrotic activities of these two stem cells were also evaluated. In addition, whether MSCs and HSCs exhibit synergistic effects in treating liver injury was studied. We hope that these findings contribute to better understanding of the interactions between stem cells and the environment that leads to homing and integration into livers.

## Materials and Methods

### Mice

Breeding pairs of C57BL/6-Ly5.1 mice and EGFP transgenic mice (C57BL/6-Ly5.2 background) were purchased from the animal center of the Fourth Military Medical University. All aspects of the animal research were approved by the Animal Care and Use Committee of the Fourth Military Medical University (Approval ID 12008) and were in compliance with Guidelines for the Care and Use of Laboratory Animals, as published by the National Academy Press.

### Isolation and Characterization of HSCs

Male EGFP transgenic mice 9 to 12 weeks old (19 to 24 grams) were used as bone marrow donors. The mice were humanely sacrificed and the bone marrow cells were flushed from the tibiae and femurs, pooled, and washed twice with Ca^2+/^Mg^2+^-free phosphate-buffered saline (PBS; Beyotime, China) containing 0.1% bovine serum albumin (BSA) (Beyotime, China). Single-cell suspensions were produced after repeated pipetting and filtering through a 50-µm nylon mesh. Bone marrow cells with densities ranging from 1.063 to 1.077 g/ml were collected by gradient separation using Nycodenz (Sigma, USA). Lineage negative (Lin^_^) bone marrow cells of the EGFP-expressing mice were prepared by incubating the cells with anti- macrophage-1(Mac-1), anti-granulocyte receptor-1 (Gr-1), anti-TER-119, anti-B220, anti-CD4 and anti-CD8 and then by removing the positive cells with immunomagnetic beads (Dynabeads M-450 coupled to sheep anti-rat IgG) (Dynal, Great Neck). The resulting Lin^_^ cells were stained with phycoerythrin (PE)-conjugated anti-Sca-1, biotin-conjugated anti-CD34, and APC-conjugated anti-c-kit and with the mouse lineage panel of antibodies, followed by streptavidin-conjugated PharRed. After the addition of propidium iodide at a concentration of 1 µg/ml, the cells were washed twice, resuspended in PBS containing 0.1% BSA, and maintained on ice for cell sorting. Five-color and cell sorting analyses were performed using a FACS Vantage (BD Biosciences, San Jose) with the appropriate isotype-matched controls. The Lin^_^ cells were enriched for the c-kit^+^/Sca-1^+^ population. Single Lin^_^, c-kit^+^, Sca-1^+^, CD34^_^ cells were deposited into round-bottomed 96-well plates using the CloneCyt system (Becton Dickinson, USA). The plates were incubated at 37°C in a humidified 5% CO_2_ atmosphere. At 20 hours after cell deposition, the wells containing single cells were marked and incubated for 7 days. We selected clones consisting of no more than 20 cells for clonal cell transplantation [18].

### Isolation, Expansion, and Phenotypic Characterization of MSCs

Bone marrow cells were collected after flushing the tibiae and femurs of 6-week-old male EGFP-expressing mice (14 to 17 grams) using sterile PBS. The cells were aspirated with a 29-gauge needle to disrupt aggregates, and then the entire aspirate was centrifuged at 2,000 rpm for 10 min. The pellet was seeded into a 25-cm^2^ culture plate with alpha-MEM (Gibco, USA) medium supplemented with 10% selected fetal bovine serum (FBS; Gibco, USA), an anti-mycotic solution (Sigma, USA) and 1% antibiotics. The non-adherent cells were removed after 72 h by changing the medium, and the medium was entirely replaced every 5 days [19]. When the foci reached conﬂuence, the adherent cells were detached with 0.25% trypsin in 2.65 mM EDTA (Sigma, USA), centrifuged, and subcultured at 7,000 cells/cm^2^. After two subcultures, the adherent cells were detached, and the cells were stained with PE -conjugated antibodies against CD29, CD90, CD34, CD105, CD80 and CD45 (Santa Cruz, USA) antigens for 30 min and then washed and analyzed using a flow cytometer (BD Biosciences, USA).

### Induction of Liver-injured Mice

Wild-type mice (9 to 12 weeks old, 19 to 24 grams) were housed in a sterile animal facility with a 12-hour dark/light cycle and free access to food and water. Advanced liver fibrosis was induced in adult female mice with intraperitoneal injections of 7 ml/kg body weight of a 1∶4 solution of CCl_4_ (Sigma, USA) and olive oil (Sigma, USA) twice a week for 3 months [18]. After final CCl_4_ injection at 3 months, the mice except the control group were randomly divided into different groups to ensure that the relatively level of liver disfunction was constant. Mice from the same cohort were randomly allocated to receive different cell stem cells via injections of the tail vein. The candidate stem cells from age- and strain-matched mice were suspended in 0.1 ml of PBS. CCl_4_ administration continued for an additional four weeks.

### Cell Transplantation

A total of 1×10^6^ cells were resuspended in 100 µL PBS and slowly infused via the tail vein (Normal, MSCs, HSCs and MSCs+HSCs treatment groups). Following transplantation, CCl_4_ was re-administered for four additional weeks (7 ml/kg body weight of a 1∶4 solution of CCl_4_ and olive oil twice a week) to enable the transplanted cells to engraft and differentiate. CCl_4_ was not administered to the normal group (n = 20) during this period. In the CCl_4_ group (n = 22), the mice were injected with CCl_4_ alone, without cell transplantation. In the MSCs group (n = 22), the mice were administered MSCs. In the HSCs group (n = 22), the mice were administered HSCs. In the MSCs+HSCs group (n = 22), the mice were administered 5×10^5^ MSCs and 5×10^5^ HSCs [17].

### Bio*-*imaging

The mice were sacrificed under deep anesthesia at 2 h, 24 h, 7 d and 28 d after transplantation. The lungs, livers, spleens and kidneys were isolated and were directly imaged by CCD with its excitation wavelength at 465/430 nm and emission filter at 560 nm with the following parameters: binning: 4, F/Stop: 1, exposure time: 1 min. The bio*-*imaging was conducted with a NightOWL imaging system using WinLight software (Berthold, Germany).

### Tissue Preparation and Confocal Microscopy

To observe the engrafted cells in the liver, the tissues were prepared as frozen sections. The livers were dissected into pieces, immediately incubated with an optimal cutting temperature compound (OCT compound, Sakura Fine technical, Japan) and held in liquid nitrogen for 20 seconds. The frozen liver sections were directly transferred to a −70°C freezer. For observing the EGFP-expressing cells in the tissue, the frozen sections were allowed to melt in distilled water for 3 min and placed on slides.

### Polymerase Chain Reaction

Genomic DNA was isolated from each liver using a lysis buffer containing 1% of 0.2 mg/ml proteinase K (Sigma, USA) and SDS followed by phenol-chloroform extraction. The genomic DNA was used to detect a Y chromosome sequence with the forward primer CTGGAGCTCTACAGTGATGA and the reverse primer CAGTTACCAATCAACACATCAC. The PCR products were fractionated by 1.2% agarose gel electrophoresis and visualized under UV illumination after staining with ethidium bromide.

### Biochemical and Liver Enzyme Assays

Serum was collected to analyze aspartate aminotransferase (AST), alanine aminotransferase (ALT), and albumin (ALB) using a chemistry analyzer (Abbott Architect c8000, USA).

### Histopathological Staining

For the histological examinations, formalin-fixed livers were dehydrated and embedded in paraffin. The liver sections were deparaffinized and stained using hematoxylin and the eosin stain or Sirius red. For immunohistochemical studies, after microwave-based antigen retrieval, the sections were treated with 0.3% H_2_O_2_ in PBS to quench the endogenous peroxidase and then incubated with 5% goat serum (Beyotime, China) to block the non-specific sites. Monoclonal primary antibodies against mouse Ki-67 (1∶100, Abcam, USA) were applied when incubating at 4°C for 12 h, followed by incubation with the corresponding secondary antibodies (Beyotime, China) at 37°C for 30 min. The specimens were then incubated with a diaminobenzidine peroxidase substrate and then subsequently counterstained with hematoxylin. For the immunofluorescence analyses, the tissue samples were fixed using 4% paraformaldehyde (Beyotime, China) and then permeabilized using 100% acetone. The samples were blocked using 5% BSA and then incubated overnight at 4°C with alpha-fetoprotein(AFP) (1∶200, Abcam, USA), ALB (1∶200, Abcam, USA), proliferating cell nuclear antigen(PCNA)(1∶500, Abcam, USA) and alpha-smooth muscle actin (α-SMA) (1∶300, Abcam, USA) antibodies diluted in antibody dilution solutions (Beyotime, China). The excess primary antibody was removed by washing five times in PBS, and the samples were incubated with a PE-conjugated secondary antibody (1∶500) at room temperature for 2 hours. The sections were incubated with 4′, 6-diamidino-2-phenylindole (DAPI; Beyotime, China) to label the nuclei. The slides were mounted in propidium iodide-containing mounting medium (Beyotime, China) for visualization using a confocal microscope (FV-1000, Olympus, Japan).

### Fluorescence-Activated Cell Sorting (FACS)

The MSCs and HSCs suspension was incubated with PE-labeled anti-mouse CXCR4 antibody (1∶500,BD Pharmingen, USA) on ice for 1 h, washed with staining buffer, and fixed with 2% paraformaldehyde. FACS data were acquired using a flow cytometer (BD Biosciences, USA) [20]. Data were analyzed using CellQuest software.

### Real-time PCR

Total RNA was isolated from fresh liver tissue and stem cells using Trizol reagent (Life Technologies, USA), and the ratio between the absorbance values at 260 and 280 nm provided an estimate of the RNA purity. Real-time PCR was performed using a one-step kit (Takara, Japan) with the following primers: α-SMA: forward: GGGAGTAATGGTTGGAATGG, reverse: GGCAGTAGTCACGAAGGAATAG; *type I collagen*: forward: AACTTTGCTTCCCAGATGTCCT, reverse: TCGGTGTCCCTTCATTCCAG; CXCR4: forward :CTCATCCTGGCCTTCATCAGC, reverse :TCAGCCAGCAGTTTCCTTGG; and β-actin: forward: TTCCTTCTTGGGTATGGAAT, reverse: GAGCAATGATCTTGATCTTC following the manufacturer’s instructions, using a Eppendorf Thermal Cycler (Takara, Japan) and using the appropriate cycle profiles.

### Enzyme-linked Immunosorbant Assays

Quantification of the mouse serum levels of nerve growth factor (NGF), hepatocyte growth factor (HGF), vascular endothelial growth factor (VEGF), IL-10, IL-6 and tumor necrosis factor-alpha (TNF-α) was determined using enzyme-linked immunosorbent assays kits per the manufacturer’s instructions (R&D Systems, USA), and the wells were read at 450 nm on an optical plate reader. Standard curves were prepared using purified cytokine standards. Each experimental sample was run in duplicate.

### Data Analyses

The data are expressed as the mean with the standard error of the mean. The statistical significances were determined using SPSS 12.0 software (SPSS, USA). The statistical significances between the control and test groups were determined using Student’s t-test. For the analyses of multiple groups, the P-values were adjusted using the Bonferroni method and a P<0.05 was used for statistical significance. All of the procedures were performed by blinded investigators.

## Results

### Characterization of EGFP-positive MSCs

**Figure 1:**
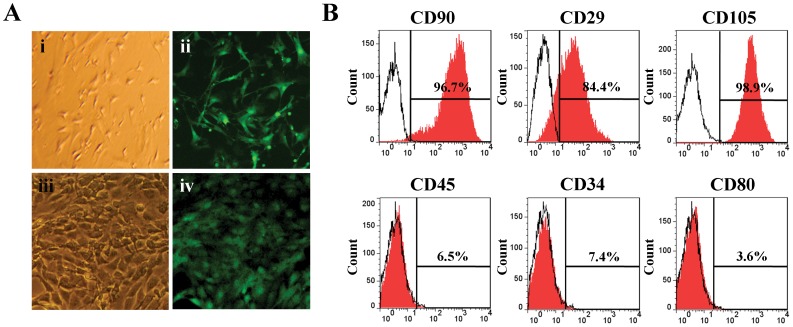
Characterization of MSCs derived from EGFP-transgenic mice. (A) The morphologies of the MSCs in the first (i and ii) and third (iii and iv) passages observed under bright field and fluorescence microscopy, respectively. (B) Surface molecule characterization of the MSCs performed by FACS analyses after incubation with PE-conjugated antibodies (CD90, CD29, CD105, CD45, CD34 and CD80).

The MSCs were isolated from the bone marrow of EGFP-transgenic mice. In the first passage, the cells derived from the donors emitted heterogeneous levels of green fluorescence when observed under the fluorescence microscope and were of various sizes, as observed in bright field. In the third passage, the EGFP signal intensity was uniform among the cells, and the cells exhibited a homogeneous morphology (Fig. 1A). Flow cytometry analyses were used to characterize the surface markers of the cultured cells. Most of the cells expressed the standard MSC surface markers, CD90 (95.2±6.1%), CD29 (85.6±3.5%) and CD105 (96.5±4.3%), whereas they were negative for CD45 (6.3±2.2%), CD34 (7.2±1.8%) and CD80 (3.8±1.2%) (Fig. 1B). MSCs were also successfully isolated for use in the following experiments.

### Detection of Donor-derived Cells

**Figure 2:**
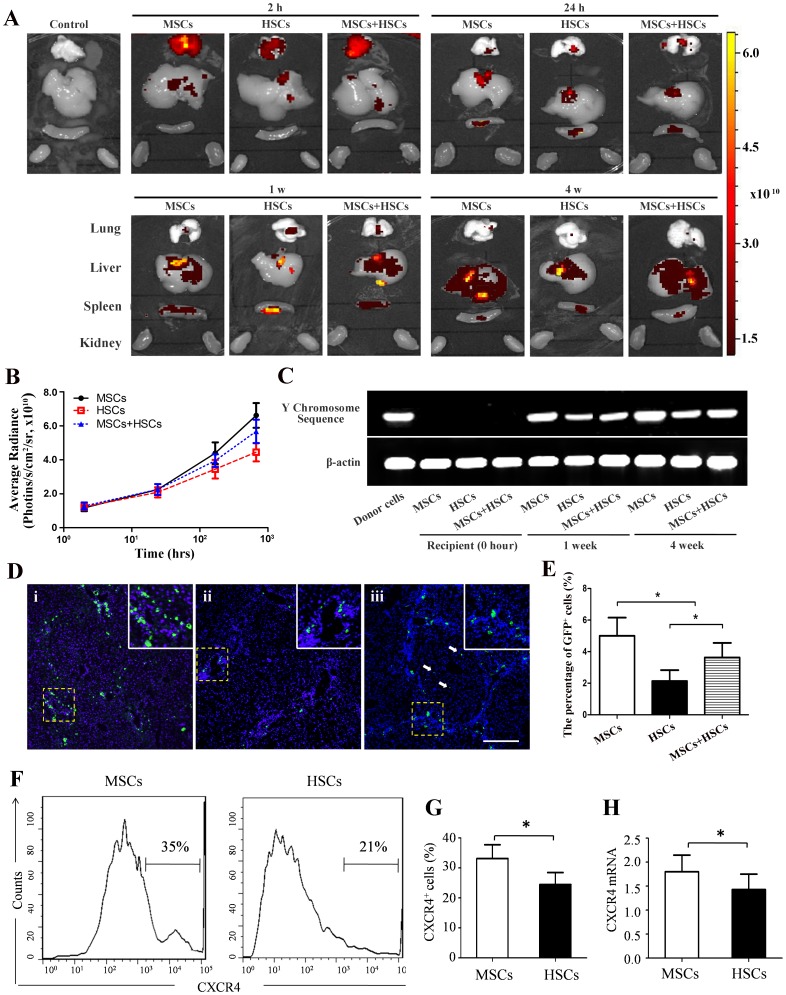
Detection of EGFP^+^ cells in recipient livers after transplantation. EGFP^+^ cells from male donors were injected into liver-injured female mice. (A) After transplantation of stem cells, the EGFP fluorescence in the lung, liver, spleen and kidney was examined using bio- imaging system. A luminescent image from red (least intense) to yellow (most intense) represents the spatial distribution of the detected photons emitted from EGFP^+^ cells within the organs. The EGFP signal was not detected in the control mice. (B) Average radiance was quantified in the liver after stem cells transplantation. (C) PCR-based detection of donor-derived cells in the livers of different recipients. (D and E) Liver sections were stained with DAPI, and the distribution of EGFP^+^ cells in the portal lobe was quantified in the different groups at 4 weeks. White arrows show the stem cells in the sinusoids (i, MSCs group; ii, HSCs group; and iii, MSCs+HSCs group). (F and G) FACS analyses of CXCR4 expression on MSCs and HSCs. (H) mRNA levels of CXCR4 on MSCs and HSCs. *P*<0.05, n = 3.

After intravenous infusion, the EGFP signals first accumulated in the lung, but by 2 h, those signals began to decrease, whereas they started to accumulate in the liver and spleen at 2 h after infusion. During the following hours to days, the EGFP signal intensity gradually increased in the liver. From 24 h to 7 d, the EGFP signal intensity gradually increased in the spleen and then decreased. The EGFP signal was barely detectable in the kidney. These trends were similar in the MSCs, HSCs and MSCs+HSCs groups (Fig. 2A). The EGFP intensity of the livers in the MSCs group was significantly higher than in the HSCs or MSCs+HSCs group (Fig. 2B). The number of homing stem cells to the CCl_4_-induced cirrhotic liver was significantly higher than that in the normal group (Fig. S1). The donor-derived signals were stronger in the livers 4 w after the cell injection than at 2 w (Fig. 2C). These results were confirmed by the percentage of EGFP-positive cells detected after nuclear staining with DAPI (Fig. 2D and 2E). From 2 w on, most of the transplanted cells were located infiltrating the areas around the liver’s portal tracts and interlobular connective tissue. Only a few of the cells migrated toward the central region of the hepatic lobes and can be detected in the sinusoids (Fig. 2D). FACS analyses of CXCR4 expression on MSCs and HSCs shows that CXCR4 expressed on MSCs (in the third passage)(33.2±8.1%) was significantly higher than HSCs(24.5±6.8%, *P*<0.05). Real-time PCR revealed that CXCR4 mRNA expression was higher in MSCs (in the third passage) than in HSCs (Fig. 2F, 2G and 2H).

### Morphology and Functional Evaluation of Injured Liver

**Figure 3:**
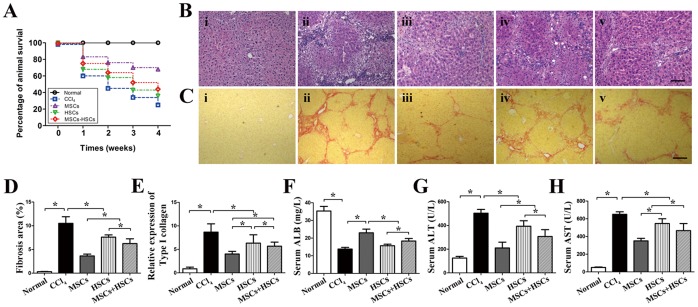
Therapeutic effects of transplanted MSCs, HSCs and MSCs+HSCs on recovery in the CCl_4_-induced injury mouse model. (A) A survival curve for the injured mice that underwent intravenous cell transplantation. Representative photomicrographs of H&E-stained (B) and Sirius red-stained (C) mouse livers from the different groups (i, normal mice; ii, CCl_4_ group; iii, MSCs group; iv, HSCs group; and v, MSCs+HSCs group).(D) Analyses of the fibrosis percentage using Image J software. (E) mRNA levels of *type I collagen*. (F, G and H) Serum ALB, ALT and AST levels in the experimental groups. *P*<0.05, n = 5, Scale bars: B = 150 µm, C = 200 µm.

**Figure 4:**
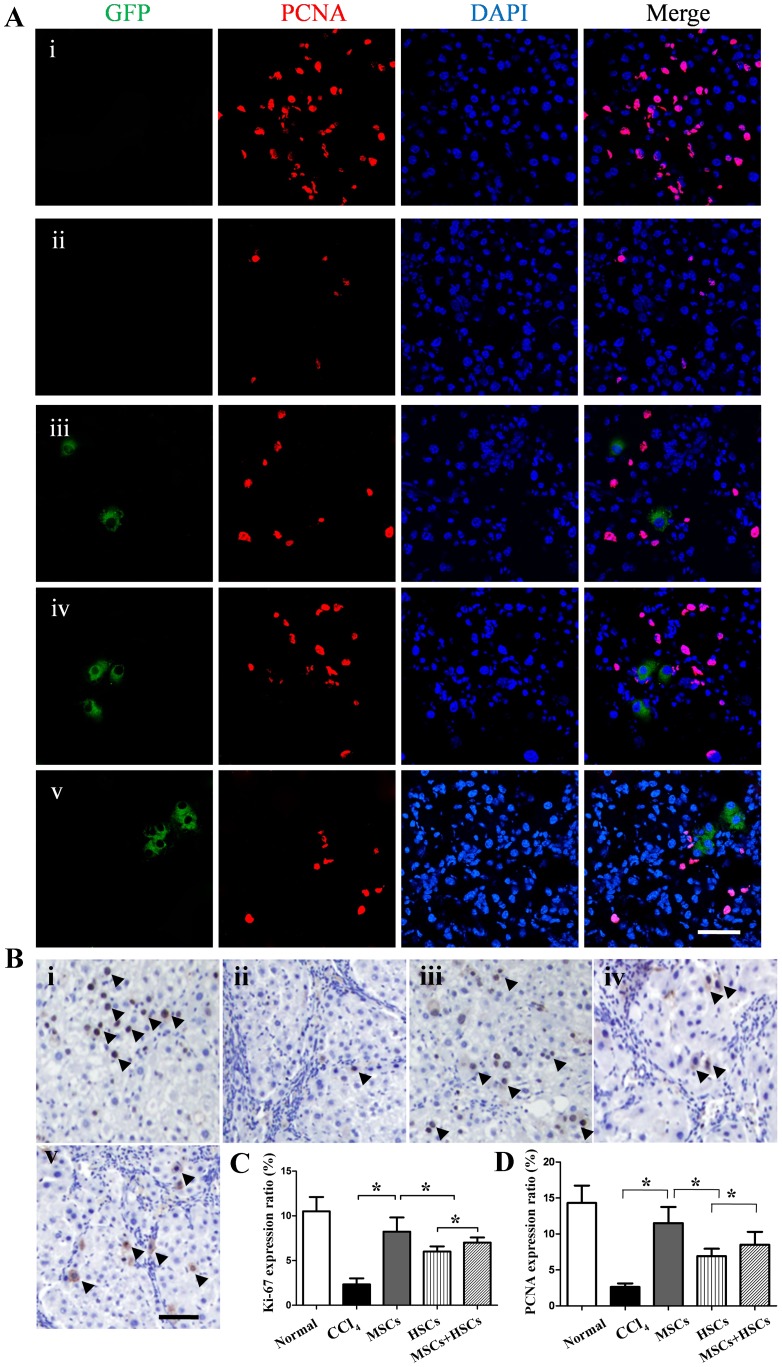
Effect of MSC, HSC and MSC+HSC transplantation on hepatocyte regeneration. (A) Immunohistochemistry analyses of PCNA expression in liver tissues (i, normal mice; ii, CCl_4_ group; iii, MSCs group; iv, HSCs group; and v, MSCs+HSCs group). (B) Immunohistochemistry analyses of Ki-67 expression in liver tissues (i, normal mice; ii, CCl_4_ group; iii, MSCs group; iv, HSCs group; and v, MSCs+HSCs group). (C) Quantitative image analyses of the percentage of Ki-67^+^ cells. (D) Quantitative image analyses of the percentage of PCNA^+^ cells. *P*<0.05, n = 5, Scale bar = 100 µm.

pone.0062426The mice in the normal group survived the observation period. The survival of the mice in the three groups that underwent stem cell transplantation was significantly higher than in the group treated with CCl_4_. The survival percentage in the MSCs group (68.2%) was significantly higher than in the HSCs (36.4%) or MSCs+HSCs (45.5%) group, while the survival percentage in the MSCs+HSCs group was significantly higher than in the HSCs group (Fig. 3A). The histological sections of the CCl_4_-injured mice demonstrated that when compared with normal mice, a large number of inflammatory cells had infiltrated the sinusoids and centrilobular regions, and the coagulation necrosis of hepatocytes was observed (H&E staining in Fig. 3B). In addition, liver fibrosis had increased significantly, characterized by fibrotic septum formation starting in the portal areas (Sirius red staining in Fig. 3C). After transplantation of MSCs, the injured livers showed maximal restoration with thinner fibrotic areas and decreased collagen depositions (3.6±0.7%). However, the fibrotic areas had decreased to a lesser extent in the mice transplanted with HSCs (7.6±0.8%) or MSCs+HSCs (6.2±1.7%, *P*<0.05) (Fig. 3D). These results were confirmed by the expression of *type I collagen* (4.0±1.1%, 6.3±3.1%, 5.7±1.5%, respectively, *P*<0.05) (Fig. 3E). Moreover, when compared with the mice in the HSCs group and the MSCs+HSCs group, the mice receiving MSCs showed the best improvement of liver function, however, liver function in MSCs group was still inferior than in the normal cohort, as demonstrated by the ALB, ALT and AST levels of the peripheral blood (Figs. 3F, 3G and 3H). To evaluate whether stem cell transplantation enhances the proliferation of hepatocytes in cirrhotic livers, the PCNA and Ki-67 expression levels were assessed by immunofluorescence and immunohistochemistry. In the MSCs-transplanted livers, the percentage of PCNA^+^ (11.5±3.4%) and Ki-67^+^ (8.2±2.7%) cells was increased significantly when compared with those of the HSCs (6.9±1.8%, 6.0±1.1%, respectively) and MSCs+HSCs (8.5±3.1%,7.2±1.9%, respectively, *P*<0.05) groups (Figs. 4A,4B,4C and 4D).

### Derivation of Transplanted Cells

**Figure 5:**
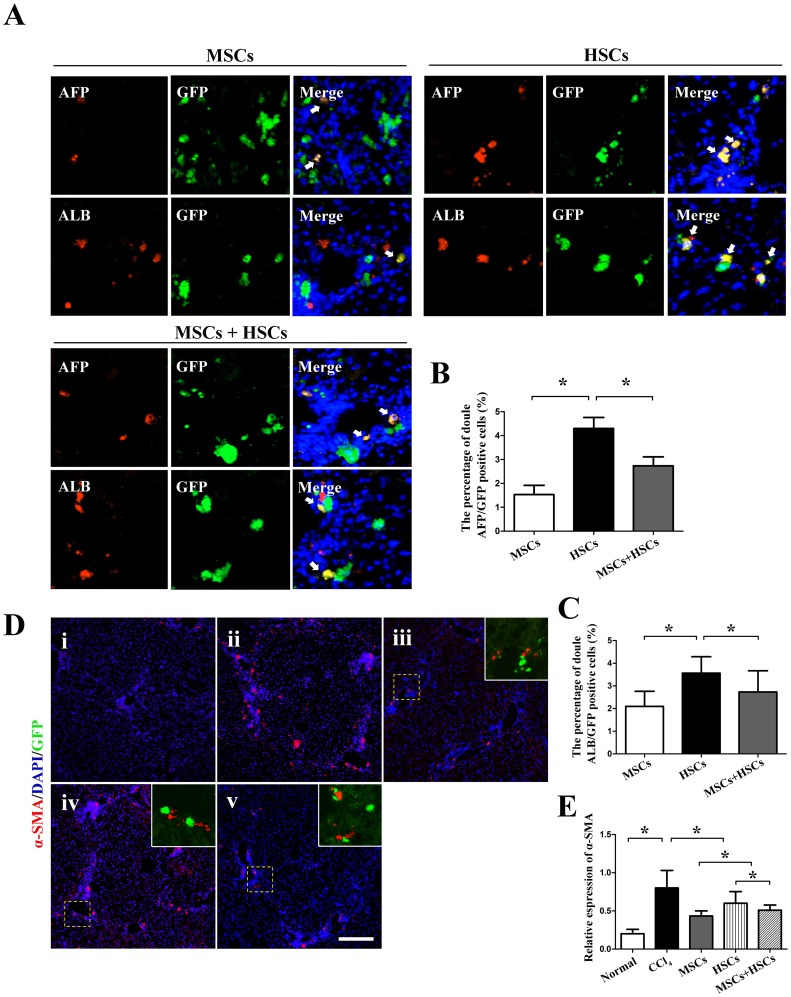
Differentiation of transplanted MSCs, HSCs and MSCs+HSCs in CCl_4_-induced injured livers at 4 weeks. The liver sections were observed under fluorescence microscopy. (A, B and C) The liver sections were co-stained with either AFP or ALB to detect the differentiation of transplanted cells (white arrowhead) in the different groups. (D) The expression of α-SMA in the livers of CCl_4_-induced injured mice in the different groups (i, normal mice; ii, CCl_4_ group; iii, MSCs group; iv, HSCs group; and v, MSCs+HSCs group). (E) mRNA levels of α-SMA. *P*<0.05, n = 5, Scale bars: A = 50 µm, D = 150 µm.

The immunofluorescence staining in the HSCs group revealed a higher percentage of double-labeled GFP^+^/AFP^+^ and GFP^+^/ALB^+^ cells in the host livers (4.3±0.6% and 3.5±0.7%, respectively), compared with the MSCs (1.4±0.5% and 2.1±0.3%, respectively) or the MSCs+HSCs (2.8±0.4% and 2.4±0.6%, respectively,*P*<0.05) groups (Figs. 5A, 5B and 5C). The expression of α-SMA was significantly decreased in the livers of mice transplanted with any cell type. The α-SMA expression in the MSCs group was significantly lower than in the other two groups (Figs. 5D and 5E). Double-labeled GFP^+^/α-SMA^+^ cells were not found in any of the groups.

### Growth Factor and Cytokine Levels in Each Group

**Figure 6:**
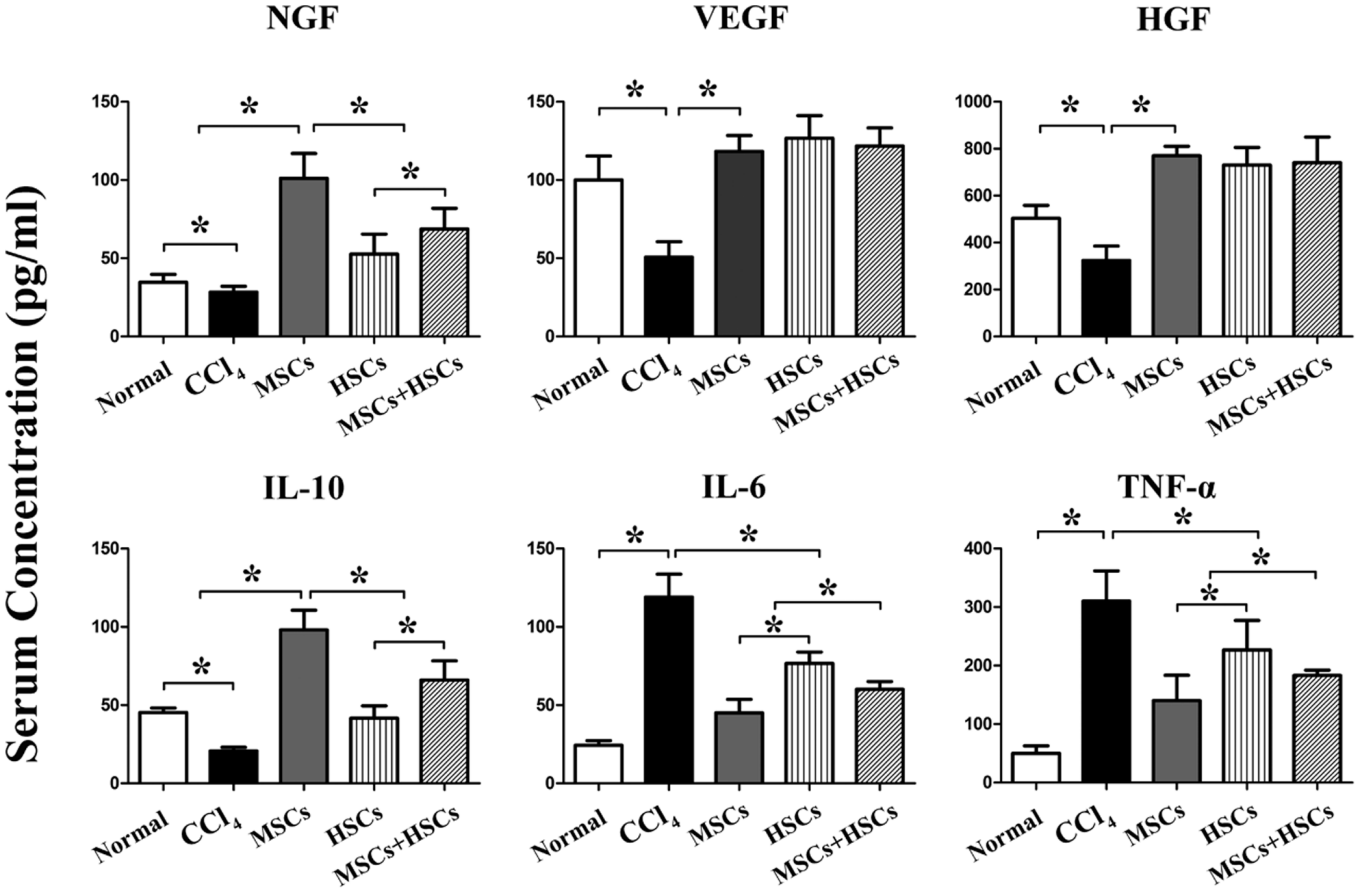
The concentration of growth factors and cytokines in each group 4 w after transplantation of MSCs, HSCs or MSCs+HSCs. *P*<0.05, n = 5. NGF, nerve growth factor; VEGF, vascular endothelial growth factor; HGF, hepatocyte growth factor; IL, interleukin; TNF-α, tumor necrosis factor-alpha.

Four weeks after cell transplantation, the serum showed a significant increase in NGF in the MSCs (101±12 pg/ml) group when compared with the HSCs and MSCs+HSCs groups (53±5 pg/ml and 69±7 pg/ml, respectively, *P*<0.05). The levels of HGF and VEGF were not significantly different among the cell transplantation groups. The expression of IL-10 in the MSCs group (98±22 pg/ml) was higher than in the other cell transplantation groups (HSCs and MSCs+HSCs groups: 42±14 pg/ml, 66±21 pg/ml, respectively) or the CCl_4_ group (21±4 pg/ml, *P*<0.05), whereas the concentration of IL-6 (45±15 pg/ml) and TNF-α (140±75 pg/ml) in the MSCs group was lower than that of HSCs (IL-6∶77±13 pg/ml, TNF-α: 227±87 pg/ml) and MSCs+HSCs (IL-6∶60±9 pg/ml, TNF-α: 183±15 pg/ml, *P*<0.05) groups (Fig. 6).

## Discussion

In this study, we conducted a comparison of MSCs and HSCs *in vivo* engraftment capacities, as well as the administration of MSCs, HSCs and MSCs+HSCs for functional liver repair using the same liver fibrosis mouse model. The major findings of our study were the following: (i) when compared with HSCs alone and a combination of HSCs and MSCs, MSCs had the greatest homing capability to the injured liver; (ii) MSCs showed the greatest capability to promote hepatocyte proliferation, inhibit hepatic stellate cell activation *in vivo* and facilitate mice survival when compared with other two groups; and (iii) synergistic effects of MSCs and HSCs were not observed in CCl_4_-induced liver injury.

Cell homing and engraftment into the host liver are integral to cell-based therapies. The mechanism that governs the recruitment of bone marrow stem cells is complicated. Several signaling pathways [21], [22], [23] and growth factors [24] have been shown to contribute to the recruitment of bone marrow cells to the injured organs. Stress in the liver results in the release of various chemokines/cytokines including stromal-derived factor-1(SDF-1) and HGF. These increase stem cells mobility through the cell surface receptors [e.g. chemokine receptor-4 (CXCR4) and hepatocyte growth factor receptor (c-met)], facilitating stem cells homing to sites of wound healing [25], [26], [27]. SDF-1-CXCR4 and HGF-c-met axes may be involved in recruitment of expanded MSCs to damaged tissues while only SDF-1-CXCR4 axes is involved in stress-induced HSCs recruitment to the injured liver [26], [27].

Previous studies regarding the homing capacity of stem cells to the injured liver have reported conflicting conclusions. Some reported that the stem cells gradually accumulated in the liver [25], [28]; however, one study demonstrated that the transplanted MSCs reached a peak amount in the liver 1 day after perfusion, and then the MSCs content gradually declined [29]. A few of the studies provided information concerning the biodistribution of the cells in major organs or compared the homing capacities of MSCs and HSCs to the injured liver.

To overcome these limitations, for the present investigation, highly purified and functionally active EGFP^+^ MSCs and HSCs isolated from bone marrow were transplanted intravenously into mice with CCl_4_-induced chronic liver injury. Our data showed that the EGFP signals were detected not only in livers but also in other tissues, such as the lungs and spleens. The distributions of these two stem cells were similar and both migrated effectively to the liver rather than to the lung, spleen and kidney in mice with CCl_4_-induced liver injury. The MSCs and HSCs gradually accumulated in the liver after they were first observed there. Furthermore, we found that MSCs exhibited a more superior homing ability to the injured liver in comparison with HSCs, which would help them exert their effects there. Meanwhile, fluorescence-activated cell sorting and Real-time PCR analyses of CXCR4 expression on both stem cells revealed that CXCR4 expression in MSCs(in the third passage)was higher than in HSCs.

As for the localization of stem cells in the liver, Sakaida et al. transplanted bone marrow cells into mice with liver fibrosis and found that the cells predominantly engrafted to the periportal area [30]. Additionally, di Bonzo et al. determined the location of human MSCs that were transplanted into mice and reported that the human cells were detected mostly around the portal tracts in injured livers [31]. Our results showed that in both injured and non-injured livers, the stem cells were detected mainly as single cells around the portal tracts and in the interlobular connective tissue. The stem cells were also detected in the sinusoids. This finding is most likely the result of the space of Disse being composed of sinusoidal endothelial cells and these cells being able release SDF-1 [32] and SDF-1can attract stem cells.

The transdifferentiation ability of bone marrow-derived stem cells into hepatocytes may play a significant role in the repair of the injured liver [13], [33], [34]. AFP and ALB typically expressed in stem cells-derived hepatocytes [28], [35], [36].However, our data showed that at 4 weeks after transplantation, the standard time of most research studies [31], the number of EGFP^+^ cells with a hepatocyte phenotype was relatively low, and the apparent number of HSCs in this model was slightly higher than that of MSCs. These cells were limited to a small portion of the total liver mass and were not sufficient to reverse the injuries (a percentage of 2.5–5% is necessary for this process [37]). Therefore, the differentiation of these two stem cells into hepatocytes cannot explain the improvement of liver function in this study.

The concept of stem cell transplantation exerting a paracrine proliferative effect on endogenous hepatocytes is gaining support. Hepatocytes in fibrotic livers have reached replicative senescence after many rounds of injury and repair, and they have reduced proliferative capacity [38], [39], [40], [41]. Stem cell infusion may increase the intrinsic ability of hepatocytes to proliferate by facilitating the breakdown of scar tissue or by the release of proliferative cytokines, thereby removing a block to proliferation [20]. Diarmaid et al. concluded that HSCs might reduce the liver injury through paracrine effects. The expression of growth factors, including HGF and VEGF, promote liver regeneration and hepatocyte proliferation [4], [42]. In addition to their differentiation to hepatocytes, MSCs infusions can also exert a paracrine proliferative effect on endogenous hepatocytes. The primary cytokines include HGF, VEGF, and NGF, which are reported to increase the intrinsic ability of hepatocytes to proliferate or to facilitate the breakdown of scar tissue [20], [43], [44], [45]. In this study, the expression levels of NGF in the MSCs group were higher than in the HSCs group, while the levels of HGF and VEGF were not significantly different in each group. We also discovered more significant hepatocyte proliferation in the MSCs group relative to the other groups. Therefore, the improvement of liver functions by MSC transplantation was partially contributed to enhancing hepatocyte proliferation.

Furthermore, the immunomodulatory properties of MSCs and HSCs can also play a significant role in the extenuation of liver injury. The local down regulation of pro-inflammatory cytokines and up regulation of anti-inflammatory cytokines, such as IL-10, after MSC transplantation has been described in kidney, lung injury and fulminant hepatic failure models [46], [47]. Pulavendran et al. found that IL-6 and TNF-α, which can be down regulated by IL-10 and are promoters of liver fibrosis, were lower in the MSCs group when compared with the control group in acute CCl_4_-induced liver injury. These authors also reported that HSCs did not seem to have any role in immunosuppression, and hence, its therapeutic application in the case of liver fibrogenic diseases is doubtful [48]. While Li et al. reported that autologous HSCs transplantation was positively correlated with the concentrations of serum IL-10 but negatively correlated with the levels of serum TNF-α in patients with type 1 diabetes [14]. Whether HSCs exhibit immunomodulatory properties in chronic CCl_4_-induced liver cirrhosis remains unclear. Our data show that the concentration of IL-10 was higher in MSCs group than in the HSCs group, although the levels in both groups were higher than that in the control group. However, the concentrations of IL-6 and TNF-α in the MSCs group were lower than those in the HSCs group. Because the proinﬂammatory phase followed by the injury leads to the induction of fibrosis, oppressing the proinﬂammatory cytokines results in the reversal of hepatic fibrosis. Thus, anti-inflammatory cytokines can reduce liver injury and then reduce hepatic fibrosis.

Several studies have demonstrated the synergistic actions of HSCs and MSCs in tissue regeneration and engineering. Moioli et al. found that the co-transplantation of HSCs and MSCs facilitated the neovascularization process in the bioengineered bone [15]. Consistently, These phenomena were also found in heart failure patients treated with a combination of HSCs and MSCs [17]. The rationale for combining these two stem cell populations came from several perspectives that include: (i) both MSCs and HSCs are mobilized after tissue injury, and thus, potentially administering both cell types may produce therapeutic synergistic activity in the injured tissue [49], [50], [51];(ii) in vitro, MSCs promote the expansion of HSCs; *in vivo*, MSCs provide a microenvironment for HSCs in both the embryonic and postnatal stage [52]. In this study, however, the synergistic actions of MSCs and HSCs had not been observed because the stem cells in liver were detected mostly as single cell which are insufficient to provide a beneficial niche for regulating the self-renewing and differentiation of HSCs.

In conclusion, these results suggest that MSCs have a greater ability to modulate chemically induced inflammation in the fibrotic liver than do HSCs. However, liver fibrosis has different causes, including alcoholic hepatitis, allograft rejection, autoimmune hepatitis and metabolic diseases. All of these disease mechanisms are quite different. In addition, the different stem cells have a variety of putative functional roles; thus, careful thought is required as to what biological action is intended from their infusion. Accordingly, further studies are needed concerning the choice of a cell therapy for specific types of liver injury.

## Supporting Information

Figure S1 **Comparison of recruitment of stem cells to the CCl_4_ -induced cirrhotic liver and the normal liver.** Average radiance was quantified in the liver after stem cells transplantation. (A,MSCs group; B, HSCs group; and C, MSCs+HSCs group). *P*<0.05, n = 3.
(TIF)
