# Supplementary figures and images for: In Vivo Tracking and Comparison of the Therapeutic Effects of MSCs and HSCs for Liver Injury
Source: PLoS One. 2013 Apr 30;8(4):e62363. doi: 10.1371/journal.pone.0062363 (PMC3640058; doi:10.1371/journal.pone.0062363)

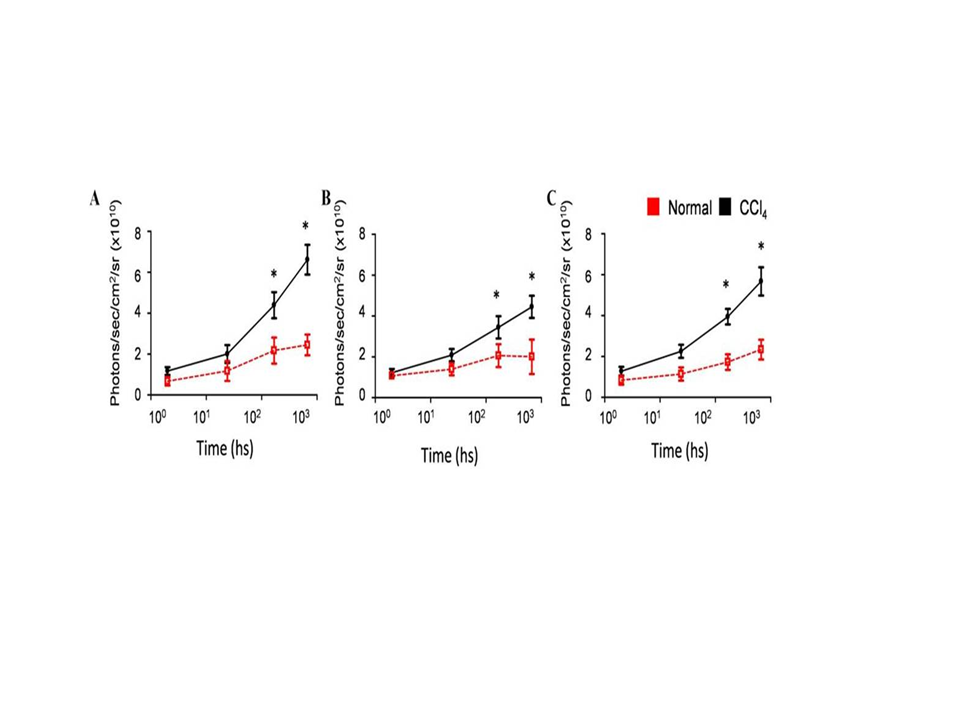

Supplement: Figure S1 — Comparison of recruitment of stem cells to the CCl4 -induced cirrhotic liver and the normal liver. Average radiance was quantified in the liver after stem cells transplantation. (A,MSCs group; B, HSCs group; and C, MSCs+HSCs group). P<0.05, n = 3. (TIF) [file pone.0062363.s001.tif]
